# COVID-19: Physical Activity, Government Restrictions, and Mental Health in the UK and Italy

**DOI:** 10.1007/s43076-023-00262-2

**Published:** 2023-04-20

**Authors:** Mikaela Sansone-Pollock, Nanette Mutrie, Cristóbal Guerra, Cristina Sechi, Silvana Mula, Clara Calia

**Affiliations:** 1 School of Health in Social Science, The University of Edinburgh, Old Medical School, Teviot Place, Edinburgh, EH8 9AG Scotland UK; 2 Moray House School of Education and Sport, The University of Edinburgh, University of Edinburgh (Holyrood Campus), Edinburgh, EH8 8AQ Scotland UK; 3 Escuela de Psicología, Facultad de Ciencias Sociales Y Comunicaciones, Universidad Santo Tomás, Santiago, Chile; 4 Department of Pedagogy, Psychology, Philosophy, University of Cagliari, Cagliari, Italy; 5 Department of Developmental and Social Psychology, Sapienza University of Rome, Rome, Italy

**Keywords:** COVID-19, Mental health, Physical activity, Government restrictions, Pandemic

## Abstract

COVID-19 restrictions could potentially induce poor mental health. This study considers opposing government restrictions on outdoor physical activity in Italy and the UK to evaluate participants’ ability to conduct physical activity, the relationship between physical activity and mental health, and whether restrictions affect mental health as mediated by physical activity. Participants from the UK and Italy self-reported physical activity before and during restrictions, sociodemographic data, and symptoms of depressions, stress, and anxiety during restrictions. Changes in physical activity were analyzed by tests of differences, and relationships between mental health, physical activity, and the effect of country restrictions were evaluated through path analysis. Two hundred sixty-four participants (UK: 57%; Italy: 43%) responded. The model (χ2(2) = .429, *p* > .05; RMSEA < .08; CFI > .90) confirmed the country’s effect on physical activity. Participants from Italy significantly decreased physical activity while the least active individuals in the UK increased activity during restrictions. Decreases in physical activity had a significant effect on increased reports of stress and depression. Physical activity did not mediate mental health within the countries. Future policies may consider ways to support individuals in maintaining physical activity habits to minimize the possibility of worsening mental health.

Since 2020, individuals have been living under variable public health measures enacted by governments around the world to slow the spread of coronavirus (COVID-19). Though vaccines have been administered to over half the world’s population by the end of 2021 (Ritchie et al., 2021), various COVID-19 restrictions remain in many countries due to continued infections and the spread of variants (Hale et al., 2021; Mancini et al., 2021). Even with hopes of an end for the current pandemic, experts are warning of subsequent pandemics and already aggregating learnings for future application (World Health Organization [WHO], 2020a)⁠. Therefore, it is critical to understand the effects of such government-imposed restrictions.

It appears that restrictions such as physical distancing and quarantine are effective at limiting new cases (Nussbaumer-Streit et al., 2020). However, these actions have required changing daily routines, limiting personal freedoms, and distancing from others. These restrictions, combined with the physical and economic reality of COVID-19, have been shown to potentially lead to poor mental health outcomes (González-Sanguino et al., 2020; Maugeri et al., 2020; Y. Zhang et al., 2020).

Physical activity (PA) is any movement of the body by skeletal muscles which requires energy expenditure (including walking, running, cycling, and exercises done inside the home; Caspersen, et al., 1985). Such activity has the potential to protect individuals from depression (Schuch et al., 2018) as well as lessen the severity of existing depressive symptoms ⁠in both clinical (Kvam et al., 2016)⁠ and non-clinical populations (Rebar et al., 2015). Similarly, PA has the potential to protect against anxiety (Schuch et al., 2019) and stress (Gerber et al., 2013) as well as reduce anxiety and stress in people with and without diagnosed disorders (McDowell et al., 2019; Moylan et al., 2013; Schultchen et al., 2019). Acknowledging these potential benefits, major governmental and organizational bodies (e.g., U.S. Center for Disease Control (CDC), WHO, and National Health Service (NHS)) have recommended regular PA specifically during this COVID-19 pandemic (NHS inform, 2020; U.S. Department of Health and Human Services, 2020; WHO, 2020b)⁠.

The WHO has highlighted the importance of being active for the benefit of both physical and mental health and makes the critical point that PA can reduce the risk of many physical health conditions such as elevated blood pressure, type 2 diabetes, and some cancers, which can “increase susceptibility to COVID-19” and elevate the risk for a more severe COVID-19 illness (Kompaniyets et al., 2021; WHO, 2020b). While it seems beneficial to promote behaviors leading to good physical health, both to counter COVID-19 susceptibility and to alleviate pressure on already-strained health services, there was previously little research which could address the relationship between PA and mental health during a pandemic. With COVID-19, research on the subject is emerging. The literature thus far concurs with non-pandemic research; higher levels of PA during COVID-19 correlate with lower reports of depression, stress, anxiety, and increased wellbeing (Jacob et al., 2020; Pieh et al., 2020)⁠. One study showed an inverse relationship between PA and life-satisfaction during restrictions but attributed this to the possibility of highly active individuals’ frustration with restrictions (S. X. Zhang et al., 2020). Importantly, the relationship between PA and mental health has previously shown to be bidirectional, with those suffering from poor mental health to be less likely to engage in PA in the first place (Azevedo Da Silva et al., 2012)⁠.

Highly relevant in this situation is not only the absolute level of PA but the relationship between changes in PA habits and mental health. COVID-19-related studies indicate that where PA decreased, compared to the pre-restriction period, or sedentary time increased, participants reported elevated depression, stress, and anxiety and decreased wellbeing (Creese et al., 2020; Faulkner et al., 2020; Meyer et al., 2020)⁠.

The present research aims to address a critical component of this relationship, the practicality of individuals engaging in regular PA under government restrictions which may specifically limit or permit PA. On March 20, 2020, Italian country-wide restrictions were broadened to include a specific ban on all outdoor PA lasting until the beginning of May (Camera dei deputati Servizio Studi, 2020). The United Kingdom (UK), not far behind Italy’s trajectory of infections (Johns Hopkins University & Medicine, 2020)⁠, imposed restrictions on March 23, 2020. Citizens were also not permitted to leave their home (except for necessities, medical appointments, etc.), but in contrast to Italy, individuals were specifically permitted to go outdoors to be physically active (Secretary of State, 2020)⁠.

This study considers the opposing COVID-19 policies of Italy and the UK during a similar period of time to evaluate the effect of restrictions on whether participants were able to engage in PA, the relationship between PA and mental health, and whether the restrictions may have had an effect on mental health as mediated by PA. It is hypothesized that decreases in PA will correlate with higher reports of stress, anxiety, and depression. Italian participants, coming from the country of stricter measures, are predicted to have decreased PA more than UK participants and therefore report lower rates of mental health during COVID-19. Finally, it is hypothesized that potential changes in PA may have a mediating role in the relationship between the country of residence and mental health; with greater restrictions, people would engage in less PA and therefore report poorer mental health.

## Design

### Participants and Procedures

A cross-sectional online survey was disseminated between end-April and end-May 2020 for UK participants (57%, *n* = 150) and until May 5, 2020 for Italian participants (43%, *n* = 114) (when restrictions on outdoor exercise were eased). Existing validated scales in English and Italian were selected to collect self-reports of PA, stress, anxiety, and depression of participants. These scales were combined with demographic questions and situational questions related to COVID-19. Following ethical approval from the School of Health in Social Science at the University of Edinburgh, the scales and questions were collated into one online survey using Qualtrics (Qualtrics, Provo, UT).

Participants were recruited online through social media and word-of-mouth. Inclusion criteria were: living in the UK or Italy, being 18 years old or older, having internet access, and speaking Italian or English. Upon entering the survey, participants were presented with information about the research procedure and purpose, as well as their rights regarding data collection. Participants consented to participate in the study then proceeded to answer the questions, which took approximately 12 minutes to complete.

## Measures

### Mental Health

#### Stress

Stress was measured using the 10-item Perceived Stress Scale (PSS-10) (Cohen & Williamson, 1988) ⁠and the validated Italian version (IPSS-10; Cronbach’s α = 0.74; Mondo et al., 2019)⁠. The PSS-10 is a reliable (Cronbach’s α = 0.78; Lee, 2012) measure to evaluate self-perceptions of stress. The PSS-10/IPSS-10 includes four positively worded items (e.g., “In the last two weeks, how often have you felt that things were going your way?”) and six negatively worded items (e.g., “How often have you felt upset because of something that happened unexpectedly?”) for participants to rate on a 5-point Likert scale. The positive items are reverse-scored and summed, resulting in a total where higher scores (0–40) indicate increased perceived stress.

#### Anxiety

The Generalized Anxiety Disorder-7 (GAD-7; Spitzer et al., 2006) ⁠is a reliable, validated, 7-item scale which evaluates self-reported anxiety symptoms (Cronbach’s α = 0.92; Spitzer et al., 2006). The Italian scale, provided by the scale authors (https://www.phqscreeners.com/), has shown good properties in Italian samples (Ivziku et al., 2019). The GAD-7 asks participants to rate their experiences in the past 2 weeks (e.g., “Feeling nervous, anxious or on edge”) on a 3-point Likert scale. The total score (0–21) is determined by summing the items; a higher score indicates higher levels of reported anxiety. Scores of 0–5, 6–10, and 11–21 represent mild, moderate, and severe anxiety symptoms respectively (Löwe et al., 2008).

#### Depression

The Patient Health Questionnaire-9 (PHQ-9; Kroenke et al., 2001) ⁠is a 9-item depression questionnaire, with good psychometric properties (Cronbach’s α = 0.86–0.89; Kroenke et al., 2001). The measure asks participants to rate their experiences in the previous 2 weeks (e.g., “Little interest or pleasure in doing things”) on a 3-point Likert scale. Scores are summed across items (0–27) with greater scores reflecting higher levels of depression. The Italian PHQ-9 was provided by the authors (https://www.phqscreeners.com/) and validated in Italian samples (Rizzo et al., 2000). Scores of 0–4, 5–9, 10–14, 15–19, and ≥ 20 represent mild, moderate, moderately severe, and severe depression symptoms respectively (Kroenke et al., 2001).

## Physical Activity

The International Physical Activity Questionnaire (IPAQ) is a validated and frequently used international measure of PA (van Poppel et al., 2010). This study used the 7-item short form (IPAQ-SF) questionnaire in English and Italian. The English version has shown reliability, *r* = 0.80, and validity* r* = 0.30 (Craig et al., 2003)⁠, and the Italian version has displayed acceptable psychometric properties (Cronbach’s α = 0.60; Mannocci et al., 2010)⁠.

The IPAQ-SF asks the number of days and average time per day engaging in vigorous activity, moderate activity, walking, and sitting. The present study prompted the first set of questions, “consider the time before restrictions from Coronavirus began,” then repeated them for “the last 7 days” (during restrictions). According to IPAQ-SF recommendations, minutes of activity were truncated to 180, those less than 10 were reduced to 0, and reported numbers were converted into a consistent measure of energy expenditure (metabolic equivalent of task (MET)-minutes per week). Category totals were summed (total MET-minutes/week), where higher scores indicated more energy expenditure, then, participants were classified into low, moderate, or high groups per IPAQ-SF guidelines (http://www.ipaq.ki.se/; Table 1 shows calculations).

**Table 1:** PA category calculations

Measure	Categories	Calculation
Physical activity level	Low	Individuals who do not meet criteria for other levels
	Moderate	≥ 3 days vigorous PA of ≥ 20 min/day OR ≥ 5 days moderate PA or walking ≥ 30 min/day OR ≥ 5 days any combination of walking, moderate, or vigorous PA ≥ 600 MET/week
	High	≥ 3 days vigorous PA ≥ 1500 MET/week OR ≥ 7 days of any combination of walking, moderate, or vigorous PA equaling ≥ 3000 MET/week
Physical activity level change	Much less exercise Less exercise Same exercise More exercise Much more exercise	Difference in PA level during restrictions compared to before restrictions For example, if a participant was “highly active” before restrictions and dropped one level down to “moderately active” during restrictions, they were coded as “less exercise” if they dropped two levels down, they were coded as “much less exercise,” and vice versa

Physical activity levels provided by IPAQ scale authors (http://www.ipaq.ki.se)

A new variable was created to compute the change in PA level before COVID-19 restrictions and during restrictions by calculating the difference between the level of PA before restrictions and the level of PA during restrictions (low (1); moderate (2); high (3)). Values were coded as follows: much less exercise (0); less exercise (1); same exercise (2); more exercise (3); much more exercise (4). If a participant increased or dropped two levels (e.g., from high activity to low activity), they were classified as “much more/less exercise” (4 or 0). If the change was only one level up or down, the participant was classified “more/less exercise” (3 or 1). If there was no change, this was coded as “same exercise” (2).

## Sociodemographic Characteristics

Sociodemographic data collected included country (UK; Italy), age range (18–34; 35–49; 50–65; more than 65), and gender (female; male). Participants were also asked their height and weight to calculate body mass index (BMI; kg/m^2^). BMI categories were determined according to NHS criteria (< 18.5 = underweight; 18.5–24.9: healthy; 25–29.9: ≤ 30: overweight; NHS, 2019). Social status was determined using the MacArthur Scale of Subjective Social Status (Cundiff et al., 2013)⁠, a one-question, valid, and pictorial scale aimed at determining indicators of self-perceived socioeconomic status (SES; Operario et al., 2004)⁠. The scale presents participants with a picture of a ladder including numbered rungs (1 = worse off to 10 = best off). Participants selected the rung number which most accurately identified where they stood on the ladder, and groups were created based on the selections: low (rungs 1–3), medium (rungs 4–7), and high (rungs 8–10) (Chen et al., 2012)⁠. Finally, participants were asked if they had access to outdoor space to be physically active.

## Statistical Analysis

The data were analyzed in two steps. First, descriptive analysis of the study variables was conducted. Then, the differences in mental health indicators (stress, anxiety, and depression) and changes in PA during COVID-19 restrictions (both countries and genders) were evaluated through chi-squared and paired and student’s *t*-tests with effect sizes reported as Cohen’s d. Spearman’s rho was used to analyze the relationship between mental health indicators and age as well as the change of PA during COVID-19. This was done using SPSS version 22.

This first part of the analysis revealed that the three mental health indicators (stress, anxiety, and depression) were strongly related to each other and that gender and age are related to mental health. Based on this, path analysis was used to evaluate a hypothesized relationship between mental health (combined in a single equation allowing for the control of covariance), PA, and the country of residence. Gender and age were set as control variables. The analysis was carried out with Mplus 7.0 (Muthén & Muthén, 1998–2012).

Given the non-normally distributed data, maximum likelihood (ML) was used with bootstrapping of 10,000 iterations (nonparametric resampling; Kenny, 2018; Mackinnon et al., 2004). Based on Schumacker and Lomax (2004)⁠, the model fit was evaluated based on the following indicators: χ2 *p* > 0.05; RMSEA ≤ 0.08; and CFI ≥ 0.90.

A post hoc power analysis using MedPower (Kenny, 2017) suggested that the sample size of 150 and 114 was adequately powered (power = 0.93 and power = 0.83, respectively) to detect the indirect effect (ab) at α = 0.05.

## Results

### Preliminary Descriptive Statistics and Bivariate Analysis

Two hundred sixty-four participants responded to the survey from the UK (57%, *n* = 150) and Italy (43%, *n* = 114) (Table 2). The majority of participants were female and 18–49 years old (83%). Between countries, there was no significant difference in age group (χ2 (3) = 6.203, *p* = 0.102), gender (χ2 (1) = 0.762, *p* = 0.383), self-perceived SES level (χ2 (2) = 2.631, *p* = 0.268), PA level before restrictions (χ2 (2) = 1.436, *p* = 0.488), or access to outdoor space to exercise (χ2 (1) = 2.308, *p* = 0.129). BMI level among UK participants was significantly higher than that of the Italian respondents; χ2 (3) = 12.71, *p* = 0.005).

**Table 2:** Sample by characteristics and country

Characteristic	Total sample		UK		Italy		*p*
Category	*N*	%	*n*	%	*n*	%	
Gender^†^							.383
Female	200	76.3%	110	74.3%	90	78.9%	
Male	62	23.7%	38	25.7%	24	21.1%	
Total	262	100.0%	148	100.0%	114	100.0%	
Age group							.102
18–34	127	48.1%	67	44.7%	60	52.6%	
35–49	91	34.5%	50	33.3%	41	36.0%	
50–64	43	16.3%	30	20.0%	13	11.4%	
≥ 65	3	1.1%	3	2.0%	0	0.0%	
Total	264	100.0%	150	100.0%	114	100.0%	
BMI (category)^†^							.005*
Underweight (< 18.5)	9	3.5%	4	2.9%	5	4.4%	
Healthy (18.5–24.9)	167	65.7%	80	57.1%	87	76.3%	
Overweight (25–29.9)	54	21.3%	39	27.9%	15	13.2%	
Obese (≥ 30)	24	9.4%	17	12.1%	7	6.1%	
Total	254	100.0%	140	100.0%	114	100.0%	
SES^†^							.268
Low (rungs 1–3)	13	4.9%	7	4.7%	6	5.3%	
Medium (rungs 4–7)	165	62.6%	100	67.1%	65	57.5%	
High (rungs 8–10)	84	31.8%	42	28.2%	42	37.2%	
Total	262	99.3%	149	100.0%	113	100.0%	
PA level before restrictions^‡^							.488
Low	45	17.0%	22	14.7%	23	20.2%	
Medium	80	30.3%	46	30.7%	34	29.9%	
High	139	52.7%	82	54.7%	57	50.0%	
Total	264	100.0%	150	100.0%	114	100.0%	
PA level during restrictions^‡^							< .001*
Low	66	25.0%	20	13.3%	46	40.4%	
Medium	88	33.3%	63	42.0%	25	21.9%	
High	110	41.7%	67	44.7%	43	37.7%	
Total	264	100.0%	150	100.0%	114	100.0%	
Outdoor space							.129
Yes	171	64.8%	103	68.7%	68	59.6%	
No	93	35.2%	47	31.3%	46	40.4%	
Total	264	100.0%	150	100.0%	114	100.0%	

*BMI*, body mass index; *SES*, socioeconomic status; *PA*, physical activity; *N/n*, frequency; *P*, significance (chi-squared test or Fisher’s exact test)

^**†**^Data total is not 264 due to missing values

^**‡**^For category calculations, see Table 1

^*^p < .05

UK and Italian participants modified PA differently during COVID-19 restrictions (Table 3; χ2 (4) = 14.89, *p* < 0.001). Although in both cases, about half of the participants altered their PA level, and a higher percentage (36%) of Italian participants decreased their usual PA compared to those in the UK (26%). There were no differences in PA changes between women and men (χ2 (4) = 4.001, *p* = 0.406).

**Table 3:** PA level change from before to during COVID-19 restrictions

PA level change	UK		Italy		Total	
	*n*	%	*n*	%	*N*	%
Much less exercise	4	2.7%	16	14.0%	20	7.6%
Less exercise	35	23.3%	25	21.9%	60	22.7%
Same exercise	83	55.3%	57	50.0%	140	53.0%
More exercise	26	17.3%	12	10.5%	38	14.4%
Much more exercise	2	1.3%	4	3.5%	6	2.3%
Total	150	100%	114	100%	264	100%

Participants in the different pre-restriction PA levels (low, moderate, high) also modified PA differently according to MET/minutes per week (Table 4). The previously high-active group in both countries significantly decreased PA during restrictions (UK: *t* (80) = 3.799, *p* < 0.001, *d* = 0.422; Italy: *t* (55) = 4.855, *p* < 0.001, *d* = 0.649) while there were no significant changes in the pre-restriction moderate groups (UK: *t* (44) =  − 1.885, *p* = 0.066, *d* = 0.281; Italy: *t* (33) =  − 0.178,* p* = 0.860, *d* = 0.030). Interestingly, the previously low-active group in the UK actually significantly increased PA during COVID-19 restrictions (*t* (21) =  − 3.499, *p* = 0.002, *d* = 0.746) while there was no significant change in this group of Italian participants (*t* (22) =  − 1.732, *p* = 0.097, *d* = 0.361).

**Table 4:** Change in PA before restrictions to during restrictions by previous level of PA

PA level before restrictions	UK			Italy		
	MET/week before	MET/week during	Δ	MET/week before	MET/week during	Δ
Low	332.25 (434)	755.75 (924)	127.5%	0 (198)	0 (1130)	0.0%
Moderate	1386.00 (951)	1473.00 (1777)	6.3%	1306.00 (1113)	1024.50 (1892)	− 21.6%
High	3466.50 (1931)	2391.00 (2350)	− 31.0%	3618.00 (1989)	1680.00 (2813)	− 53.6%

Participants in the high-active group before restrictions (who significantly decreased PA during restrictions, mentioned above) also reported significantly more instances of higher depression (χ2 (4) = 9.752, *p* = 0.045). When stratified by country, this was only significant when considering the high-active Italian participants (χ2 (4) = 12.243, *p* = 0.016).

Regarding mental health, Table 5 shows the minimum and maximum values, the mean (and standard deviation), and skewness and kurtosis. Italian participants reported slightly higher values of stress (17.56 vs. 16.82; *t* (257) =  − 0.875, *p* = 0.382, *d* = 0.115), anxiety (8.12 vs. 7.33; *t* (257) =  − 1.120, *p* = 0.264, *d* = 0.140), and depression (8.22 vs. 8.15; *t* (256) =  − 0.092, *p* = 0.927, *d* = 0.011) than those of the UK, but not to a significant degree. Women reported significantly higher levels than men: stress (17.84 vs. 14.60; *t* (255) = 3.406, *p* < 0.001, *d* = 0.497), anxiety (8.25 vs. 5.46; *t* (255) = 3.471 *p* < 0.001, *d* = 0.509), and depression (8.72 vs. 6.19; *t* (254) = 3.096, *p* = 0.002, *d* = 0.452).

**Table 5:** Descriptive statistics and correlations between mental health indicators

	Minimum / maximum	Mean (SD)	Skewness	Kurtosis	GAD	PHQ	Age	PA change
PSS-10 total	1–36	17.14 (6.71)	.01	− .58	.82**	.79**	− .30**	− .13*
GAD-7 total	0–21	7.68 (5.67)	.71	− .42	-	.82**	− .33**	− .13*
PHQ-9 total	0–24	8.18 (5.73)	.62	− .38		-	− .31**	− .19**

*Perceived Stress Scale (PSS-10)*, range 0–40; *Generalized Anxiety Disorder (GAD-7)*, range 0–21; *Patient Health Questionnaire (PHQ-9)*, range 0–27. Spearman’s rho

^*^p < .05; **p < .01

Positive and significant relationships were observed between the three mental health measures (Table 5). For example, increased reports of depression were significantly related to increased reports of anxiety and stress. Table 6 shows that changes in PA levels during the pandemic were inversely associated with stress, anxiety, and depression, along with age (the younger participants and those who decreased PA during restrictions showed higher levels of stress, anxiety, and depression; Table 6).

**Table 6:** Regression coefficients in the proposed path analysis

Variable	*B*	*B*	95% CI
Stress			
Age	− 2.56**	− .30**	− 3.49, − 1.63
Gender (1 = female; 2 = male)	− 2.72**	− .17**	− 4.43, − 1.01
Country (1 = UK; 2 = Italy)	.04	0	− 1.51, 1.59
PA change	− 1.05*	− .13*	− 1.94, − .15
* R*^2^	.15		
Anxiety			
Age	− 2.16**	− .30**	− 2.88, − 1.44
Gender (1 = female; 2 = male)	− 2.50**	− .19**	− 3.87, − 1.12
Country (1 = UK; 2 = Italy)	.20	.02	− 1.08, 1.49
PA change	− .58	− .09	− 1.37, .21
* R*^2^	.15		
Depression			
Age	− 2.26**	− .31**	− 3.01, − 1.50
Gender (1 = female; 2 = male)	− 2.08**	− .16**	− 3.51, − .66
Country (1 = UK; 2 = Italy)	− .58	− .05	− 1.89, .74
PA change	− 1.38**	− .21**	− 2.16, − .60
* R*^2^	.17		
PA level change			
Country (1 = UK; 2 = Italy)	− .24*	− .14*	− .45, − .02
* R*^2^	.02		

^*^p < .05; **p < .01

Preliminary bivariate analysis was carried out to determine whether to include age and gender as control variables in the model while allowing for the necessary degrees of freedom to calculate adjustment indicators. Gender was associated with mental health in that women reported significantly higher levels than men in stress (17.84 vs. 14.60; *t* (255) = 3.406, *p* < 0.001, *d* = 0.497), anxiety (8.25 vs. 5.46; *t* (255) = 3.471 *p* < 0.001, *d* = 0.509), and depression (8.72 vs. 6.19; *t* (254) = 3.096, *p* = 0.002, *d* = 0.452). However, gender was not related to the change in PA (*X*^2^_(4)_ = 4.001; *p* = 0.41). Age was inversely associated with stress, anxiety, and depression (see Table 6), but also not with changes in PA (*rho* = 0.06; *p* = . 37). Therefore, gender and age were included as control variables for mental health but not for change in PA.

## Evaluation of the Explanatory Model (Multivariate Analysis)

The model evaluates how mental health (stress, anxiety, and depression) is different in each country (with more restrictive COVID-19 regulations in Italy), and how this effect may be mediated by changes in the practices of PA. Based on the results of the preliminary analyses, gender and age were included as control variables.

**Fig. 1:**
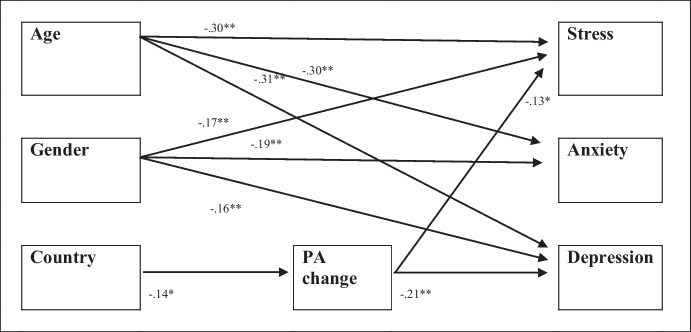
Path of predictive relations. *p < .05; **p < .01. Note: Non-significant effects are excluded

The results highlighted in Table 6 confirm the observations from the preliminary analyses. Gender and age showed a direct effect on stress, anxiety, and depression (women and younger people reported greater symptoms). The country of residence did not show a direct effect on mental health indicators. However, the country of residence related to changes in PA (in Italy, PA decreased to a greater extent than in the UK) and higher symptoms of stress and depression were reported for participants who decreased PA. There was no meditational effect of PA change regarding the country’s effect on symptoms (*B* indirect effect = 0.02, *p* = 0.147 for stress; *B* indirect effect = 0.03, *p* = 0.072 for depression). The model has a good fit (χ2 (2) = 0.429, *p* > 0.05; RMSEA < 0.08; TLI > 0.90, CFI > 0.90, and SRMR < 0.08) and explains 15% of the variance of stress, 15% of the variance of anxiety, and 17% of the variance of depression. Figure 1 shows the standardized coefficients which are statistically significant*.*

## Discussion

This study considered two countries with differing COVID-19 restrictions to evaluate the relationship between changes in physical activity (PA) habits, mental health (stress, anxiety, and depression), and the influence of government-imposed restrictions. It was found that participants from Italy, the country with more restrictive measures, experienced a significantly greater decrease in PA than those in the UK, a country which allowed outdoor PA. The model showed greater decreases in PA during COVID-19 restrictions to have a significant effect on the increased reports of stress and depression. The country of residence, and therefore possibly the restrictions, had a direct effect on the change in PA during COVID-19. The model did not show PA to be a significant mediator of the relationship between country and mental health; however, the aforementioned direct relationships between country and PA and PA and mental health are informative with regard to the effects and influences of restrictions.

The finding that decreases in PA related to poorer mental health concurs with other COVID-19 reports (Creese et al., 2020; Faulkner et al., 2020)⁠. This also supports non-pandemic research which has shown PA withdrawal to negatively affect mental health (Weinstein et al., 2017)⁠. The decrease in PA among participants in this study occurred primarily among those who were previously the most active in both countries. This is unsurprising and also in line with recent studies (Meyer et al., 2020)⁠. The finding that previously highly active participants in Italy, and not those in the UK, reported significantly higher depression may support the finding of Zhang and colleagues (2020) that very active individuals could feel particularly frustrated with restrictions.

The present research also found that as a group, participants in the UK who engaged in PA at the lowest level before restrictions significantly increased their MET during COVID-19 restrictions, whereas, although Italian participants in this category also experienced an increase, it was not to the same degree, nor significant. These UK participants went from not meeting PA guidelines prior to restrictions into the category of meeting recommended MET per week (U.S. Department of Health and Human Services, 2018). This finding further alludes to the potential effect of COVID-19 restrictions and highlights a possibility to increase PA in a group of citizens, potentially having a positive impact on mental health.

The increase in PA from the previously least active participants could have been due to fewer competing interests for time and attention as many individuals were no longer commuting to work, and entertainment venues were closed. In the UK, citizens were also explicitly permitted to exercise, with this messaging clearly included within the restrictions (Secretary of State, 2020). Exercise was advocated as one of the only legitimate reasons for leaving the home and one of the only options for individuals to go outside and get fresh air if they desired.

These findings suggest that it would benefit the mental health of citizens who are placed under restrictions to be supported in avoiding decreases in PA routines. This could mean allowing outdoor PA, permitting fitness locations to remain open when possible or encouraging them to move regular activities outdoors, and messaging citizens about the opportunity to engage in PA during restrictions. Additionally, there may be a unique opportunity to increase PA in previously inactive individuals through messaging and policy which explicitly allows people to leave home for physical activity. These accommodations for PA would be likely have the additional effect of reducing the risk of physical health conditions (e.g., type 2 diabetes, high blood pressure, various cancers) during restrictions and beyond (Piercy et al., 2018; WHO, 2020b).

This study had several limitations. First, the cross-sectional study design cannot confirm causality. Second, this research required reliance on self-reports for PA and mental health, and there was no baseline data for this sample. The self-reported PA from the period before restrictions could have suffered recall bias. Third, as participants were recruited conveniently, this may have led to sample skews, including attracting a highly active sample (around 83% of the participants in this research were “active” before restrictions, whereas the NHS (2019)⁠ estimates only 66% of the general population that is “active”). The participants were also overwhelmingly female (76.3%), and there was a difference in the UK and Italian samples in terms of BMI which could have affected participation in PA (Besson et al., 2009). This study also had strengths including collecting data from participants in two different countries at the same time where COVID-19 restrictions differed in the permissibility of outdoor PA. This study also utilized existing and established measures to readily contribute to the growing body of research on PA and mental health during a pandemic.

## Conclusion

Government-imposed pandemic restrictions which allow individuals to maintain PA routines, or even improve PA habits, might reduce the risk of deteriorating mental health. As COVID-19 restrictions have been ongoing and experts are already preparing for future pandemics (WHO, 2020a)⁠, this research adds to a growing body of literature which may be referenced in developing public messaging and policy to minimize the mental health impacts of restrictions and the virus overall.

## Data Availability

The data and material are available upon reasonable request.
